# Corallopyronin A exhibits potent activity against staphylococci including MRSA and isolates from prosthetic infections

**DOI:** 10.1007/s15010-026-02760-8

**Published:** 2026-03-12

**Authors:** Jesenko Karačić, Miriam Grosse, Kenneth Pfarr, Andrea Schiefer, Tanja Schneider, Achim Hoerauf, Sabina Karačić, Marijo Parčina, Gunnar Hischebeth, Frank Sebastian Fröschen, Gabriele Bierbaum

**Affiliations:** 1 Institute of Medical Microbiology, Immunology and Parasitology, University Hospital Bonn, Venusberg‐Campus 1, 53127 Bonn, Germany; 2 German Center for Infection Research (DZIF), Partner Site Bonn-Cologne, Bonn, Germany; 3 Department of Microbial Drugs, Helmholtz Centre for Infection Research, 38124 Brunswick, Germany; 4 Institute for Pharmaceutical Microbiology, University Hospital Bonn, 53115 Bonn, Germany; 5 Department of Orthopaedics and Trauma Surgery, University Hospital Bonn, 53127 Bonn, Germany

**Keywords:** Corallopyronin A, Staphylococci, MRSA, Antibiotic interactions, SCV

## Abstract

**Purpose:**

This study evaluates the in vitro antimicrobial activity of Corallopyronin A (CorA) against a diverse collection of *Staphylococcus aureus* and coagulase-negative staphylococci (CNS), comprising both laboratory strains and clinical isolates. The dataset includes methicillin-resistant and methicillin-susceptible strains, as well as small colony variants (SCVs), to assess its therapeutic potential in staphylococcal infections.

**Methods:**

A total of 116 staphylococcal strains, comprising clinical isolates and laboratory strains, were subjected to minimum inhibitory concentration (MIC) testing. Minimum bactericidal concentrations (MBCs) were determined for a subset of 70 strains. Time-kill assays were conducted for five *S. aureus* strains using 4 × MIC of CorA. Additionally, checkerboard assays were performed with 11 antibiotics to evaluate potential additive or synergistic interactions.

**Results:**

CorA demonstrated potent antimicrobial activity with MIC values ranging from 0.125 to 2 mg/L. The MIC_90_ was 0.5 mg/L for *S. aureus* and 1 mg/L for CNS. Methicillin-resistant strains exhibited significantly higher susceptibility than methicillin-sensitive strains. Time-kill assays revealed a reduction of 1.5-3 log_10_ CFU/mL in viable counts within 24 h. Minimum bactericidal concentration testing showed bactericidal activity in a subset of strains, occurring in 71% of CNS isolates and 34% of *S. aureus* strains, while the remaining *S. aureus* and CNS isolates displayed a bacteriostatic response. Checkerboard assays indicated additive interactions with glycopeptides, including dalbavancin and oritavancin.

**Conclusions:**

CorA shows strong in vitro activity against a broad range of staphylococcal strains, particularly methicillin-resistant isolates. Its additive effects with clinically relevant antibiotics further support its potential in combination therapy for the treatment of resistant staphylococcal infections.

**Supplementary Information:**

The online version contains supplementary material available at 10.1007/s15010-026-02760-8.

## Introduction

*Staphylococcus aureus* is a major human pathogen responsible for many infections, ranging from superficial skin infections to severe invasive diseases. Among these, infections associated with implanted medical devices, such as osteomyelitis and periprosthetic joint infections (PJIs), pose significant clinical challenges [1–4]. These infections are difficult to manage due to the ability of *S. aureus* to persist within host tissues and form biofilms, which protect bacterial cells from both the host immune response and antimicrobial agents. In addition to the clinical impact of *S. aureus*, the increasing prevalence of methicillin-resistant *S. aureus* (MRSA) and the emergence of small colony variants (SCVs)-which exhibit slow growth and increased antibiotic resistance-further complicate the difficulty in treating these infections [5–9].

Corallopyronin A (CorA), a naturally occurring antibiotic produced by the myxobacterium *Corallococcus coralloides*, offers a promising alternative for addressing these challenges [10]. Unlike traditional antibiotics such as rifampicin [11], CorA targets bacterial RNA polymerase (RNAP) by binding to the switch region, disrupting its ability to open the clamp and initiate transcription [12–15]. In contrast, rifampicin binds in the DNA-binding channel, close to the active site of RNA-polymerase. These differences in binding sites avoid the development of cross-resistance with rifampicin; resistance to rifampicin is a significant limitation in current treatments for *S. aureus* infections [16, 17].

First investigations have demonstrated the broad-spectrum activity of CorA against Gram-positive pathogens, including MRSA and coagulase-negative staphylococci (CNS), as well as intracellular bacteria such as *Chlamydia trachomatis*, *Neisseria gonorrhoeae*, *Orientia tsutsugamushi*, and *Wolbachia*, highlighting its versatility [18–23]. CorA has successfully passed in vitro and in vivo non-GLP ADMET studies and is in preclinical development for the treatment of filarial nematode infections, due to its activity against the essential endosymbiont *Wolbachia* [24].

Because *S. aureus* is a major contributor to periprosthetic joint infections (~ 60%) (PJIs), CorA can potentially be a promising therapeutic option for treating these infections. Given the increasing prevalence of antimicrobial resistance and the persistence of biofilm-associated infections, this study investigated the antimicrobial potential of CorA against a broad range of *S. aureus* strains and coagulase-negative staphylococci (CNS), including strains from prosthesis and soft tissue infections.

The dataset comprises both clinical and laboratory isolates, including type strains, methicillin-resistant and methicillin-sensitive strains, a selection of epidemic MRSA strains of different clonal complexes, including livestock-associated MRSA, vancomycin-intermediate *S. aureus* (VISA), and well-characterized laboratory strains used for antibiotic screening and testing. Stable small colony variants (SCVs), which are associated with persistent and chronic infections, were used to assess the efficacy of CorA against these difficult-to-treat phenotypes. The clinical isolates were isolated either from tissue samples or blood cultures.

Minimum inhibitory concentrations (MICs) and minimum bactericidal concentrations (MBCs) were determined to evaluate the antimicrobial activity of CorA across this diverse strain set. Furthermore, time-kill assays were performed to assess dynamic bactericidal effects over time.

Finally, checkerboard assays were implemented to assess the activity of CorA in combination with 11 antibiotics, evaluating potential additive or synergistic interactions, particularly with agents commonly used in the treatment of staphylococcal infections. These comprehensive analyses provide new insights into the therapeutic potential of CorA, particularly in addressing the growing threat of antimicrobial resistance in staphylococcal pathogens.

## Material and methods

### Strains

All tested strains, their characteristics and their MICs are presented in Tables 1, 2, 3, 4.

**Table 1:** Overview of MRSA laboratory and clinical isolates used in this study, including MIC values of Corallopyronin A (CorA), antimicrobial resistance profiles, isolate origin, and sequence type (ST/CC)

Nb	*S. aureus* Strains	MIC CorA (mg/L)	Strain collection/reference	Additional resistances of clinical isolates	Isolate origin	CC/ ST-type
MRSA						
1	*S. aureus* ATCC 33591	0.5	ATCC	Ery, Cli, Tet	type strain, 1884 pleural fluid	ST239
2	*S. aureus* LT216/12	0.5	clinical isolate, BN TL	Dox, Sxt	nose	ST398
3	*S. aureus* LT181/12	0.5	clinical isolate, BN TL	Mox, Sxt	throat	ST88
4	*S. aureus* 1450/94	0.5	RKI, Wernigerode	Gen, Ery, Cli, Tet, Sxt, Rif, Cip (H481N, L875S, V798A RpoB)	PFGE control strain	ST247
5	*S. aureus* 1000/93	0.5	RKI, Wernigerode	Gen, Ami, Net, Ery, Tet, Cip	PFGE control strain	ST254
6	*S. aureus* 825/96	0.5	RKI, Wernigerode	Ery, Cip	PFGE control strain	CC45
7	*S. aureus* 635/93	0.5	RKI, Wernigerode	Gen, Ami, Net, Ery, Cli, Imi, Sxt, Fos, Cip	PFGE control strain	ST239
8	*S. aureus* 831/96	0.5	RKI, Wernigerode	Gen, Ery, Clin, Cip	PFGE control strain	ST228
9	*S. aureus* 252, NCTC 13277	0.5	NCTC	Amk, Ery, Cli, Kan, Cip, Sxt	wound	ST36
10	*S. aureus* USA 300 Ref 2	0.5	RKI, Wernigerode	Ery, Lev	PFGE control strain	ST8
11	*S. aureus* USA100_NRS382	0.5	NARSA	Oxa, Lev,Ery,Cli	PFGE control strain	ST5
12	*S. aureus* LT03/2011	0.5	clinical isolate, BN TL	Ery, Cli, Cip, Mox	tracheal secretion	ST225
13	*S. aureus* LT270/12	0.5	clinical isolate, BN TL	Ery, Cli	wound	CC398
14	*S. aureus* LT137/93A	0.5	Kleinert et al. 2016 [32]	Gen, Ery, Cli, Tet, Sxt, Rif, Cip (H481N, L875S, V798A, S529L RpoB), VISA	tracheal secretion	ST247
15	*S. aureus* LT380/12	0.5	clinical isolate, BN TL	Ery, Cli, Sxt, Dox	throat	CC398
16	*S. aureus* LT428/12	0.5	clinical isolate, BN TL	Ery, Cli, Sxt, Dox	nose	CC398
17	*S. aureus* LT160/12	0.5	clinical isolate, BN TL	Dox	lung	CC398
18	*S. aureus* LT283/12	0.5	clinical isolate, BN TL	**-**	nose	CC398
19	*S. aureus* LT429/12	0.25	clinical isolate, BN TL	Ery, Cli, Dox	tissue	CC1
20	*S. aureus* LT234/12	0.25	clinical isolate, BN TL	Ery, Cli, Cip	nose	CC22
21	*S. aureus* USA300_FPR3757	0.25	Diep et al. 2006 [33]	Ery, Cli, Cip, Tet, Mup	abscess	CC8
22	*S. aureus* DSM 11822	0.25	DSMZ	Gen, Lev, Cli, Tet	lab strain*	CC8/239
23	*S. aureus* Mu50	0.25	Kuroda et al. 2001 [34]	Ery, Cli, Tet, Ami, Spc, Rif, VISA	wound	ST5
24	*S. aureus* LT174/12	0.25	clinical isolate, BN TL	Ery, Cli, Cip, Sxt	nose	ST225
25	*S. aureus* LT425/12	0.25	clinical isolate, BN TL	**-**	nose	ST80
26	*S. aureus* Ia48	0.25	Begic et al. 2009 [30]	Ery, derived from *S. aureus* COL	stable SCV, lab strain	ST250
27	*S. aureus* COL	0.125	Steven et al. 2025 [35]	Tei, Tet	lab strain	ST250

Antibiotic abbreviations: *Oxa* oxacillin, *Ery* erythromycin, *Cli* clindamycin, *Tet* tetracycline, *Sxt* trimethoprim-sulfamethoxazole, *Gen* gentamicin, *Amk* amikacin, *Fos* fosfomycin, *Cip* ciprofloxacin, *Rif* rifampicin, *Van* vancomycin, *Tei* teicoplanin, *Dox* doxycycline, *Mup* mupirocin, *Lev* levofloxacin, *Pen* penicillin, *BN TL* Bonn Typing Laboratory

Type (*) or quality control strains (**) are marked with asterisks

**Table 2:** Overview of methicillin-sensitive *Staphylococcus aureus* (MSSA) strains with available sequence type (ST/CC). MIC values for CorA, resistance data, strain origin, and classification as clinical or laboratory strains are presented

Nb	*S. aureus* Strains	MIC CorA (mg/L)	Strain collection/reference	Additional resistances of clinical isolates	Isolate origin	CC/ST-type
MSSA						
1	*S. aureus* ATCC 13709	1	ATCC		lab strain	ST707
2	*S. aureus* RN4220	1	Ko et al. 2025 [36]	-	lab strain	ST8
3	*S. aureus* DSM 18586, ATCC 14458	1	DSMZ	Pen, Tet	feces	ST250
4	*S. aureus* 6850, ATCC 53657	0.5	ATCC	Pen	joint infection	ST50
5	*S. aureus* ATCC 25923	0.5	ATCC	-	lab strain	ST243
6	*S. aureus* COWAN1, ATCC 12598	0.5	ATCC	-	tissue	ST8
7	*S. aureus* ATCC 29213, DSM 2569	0.5	DSMZ	-	wound	ST5
8	*S. aureus* HG001	0.25	Herbert et al. 2010 [37]	-	lab strain	ST8
9	*S. aureus* DSM 28763 ECC5055	0.25	DSMZ	-	wound	ST8
10	*S. aureus* Newman ATCC25904	0.25	ATCC	-	lab strain	ST254
11	*S. aureus* Wood 46 DSM 20491	0.25	DSMZ	-	lab strain	ST97
12	*S. aureus* SG511-Berlin	0.25	Dietrich et al. 2021[38]	-	lab strain	ST30
13	*S. aureus* SA113, NCTC 8325	0.25	NCTC	-	lab strain	ST8
14	*S. aureus* DSM 20231	0.25	DSMZ	-	type strain, pleural fluid	ST8
15	*S. aureus* 15981	0.25	Valle et al. 2003 [39]	-	tissue	ST50
16	*S. aureus* I10	0.25	von Eiff et al. 1997, derived from *S. aureus* NCTC 8325–4 [29]	Ery	Stable SCV, lab strain	ST8

Antibiotic abbreviations: *Ery* erythromycin, *Tet* tetracycline, *Pen* penicillin

**Table 3:** Overview of MRSA (A) and MSSA (B) clinical isolates without available sequence type (ST/CC) data. The table presents MIC values for CorA, antibiotic resistance profiles, and strain origin

Nb	*S. aureus* Strains	MIC CorA (mg/L)	Strain Collection/reference	Additional resistances of clinical isolates	Tissue isolates
A) MRSA					
1	*S. aureus* R1	0.5	clinical isolate UKB	Sxt, Tri	joint infection
2	*S. aureus* R2	0.5	clinical isolate UKB	Fus	abdomen
3	*S. aureus* R3	0.5	clinical isolate UKB	Ery, Cli, Lev	joint infection
4	*S. aureus* R4	0.25	clinical isolate UKB	Ery, Cli, Lev	wound after amputation
5	*S. aureus* R5	0.25	clinical isolate UKB	**-**	sonication
6	*S. aureus* R6	0.25	clinical isolate UKB	Tet, Ery, Cli	amputated tissue
7	*S. aureus* R7	0.25	clinical isolate UKB	Tet, Ery, Cli	bone tissue
8	*S. aureus* R8	0.25	clinical isolate UKB	Ery, Cli, Lev	sonication
9	*S. aureus* R9	0.25	clinical isolate UKB	Tet, Ery	sonication
10	*S. aureus* R10	0.25	clinical isolate UKB	Ery, Cli	bone tissue
11	*S. aureus* R11	0.25	clinical isolate UKB	Lev	joint tissue
12	S. *aureus* R12	0.25	clinical isolate UKB	**-**	prosthesis inf
13	*S. aureus* R13	0.25	clinical isolate UKB	Dap, Lev	abdomen
14	*S. aureus* R14	0.25	clinical isolate UKB	**-**	sonication

Nb	*S. aureus* Strains	MIC CorA (mg/L)	Strain Collection/reference	Additional resistances of clinical isolates	Source
B) MSSA					
1	*S. aureus* S1	0.5	clinical isolate UKB	Pen	blood culture
2	*S. aureus* S2	0.5	clinical isolate UKB	Pen	blood culture
3	*S. aureus* S3	0.5	clinical isolate UKB	Pen	blood culture
4	*S. aureus* S4	0.5	clinical isolate UKB	Pen	blood culture
5	*S. aureus* S5	0.5	clinical isolate UKB	Pen	blood culture
6	*S. aureus* S6	0.5	clinical isolate UKB	-	blood culture
7	*S. aureus* S7	0.5	clinical isolate UKB	-	blood culture
8	*S. aureus* S8	0.25	clinical isolate UKB	-	blood culture
9	*S. aureus* S9	0.25	clinical isolate UKB	Pen	blood culture
10	*S. aureus* S10	1	clinical isolate UKB	Pen, Cli, Rif	sonication
11	*S. aureus* S11	0.25	clinical isolate UKB	Pen	sonication
12	*S. aureus* S12	0.25	clinical isolate UKB	Pen, Ery, Cli	sonication
13	*S. aureus* S13	0.5	clinical isolate UKB	Pen	sonication
14	*S. aureus* S14	0.5	clinical isolate UKB	Pen	sonication
15	*S. aureus* S15	0.125	clinical isolate UKB	-	sonication
16	*S. aureus* S16	0.125	clinical isolate UKB	-	sonication

Antibiotic abbreviations: *Sxt* trimethoprim-sulfamethoxazole, *Tet* tetracycline, *Ery* erythromycin, *Cli* clindamycin, *Lev* levofloxacin, *Pen* penicillin, *Dap* daptomycin, *Rif* rifampicin. “Sonication” marks strains that were isolated after sonication of materials obtained from prosthesis infections; the other strains are derived from infected tissue, as indicated or blood culture

**Table 4:** Overview of coagulase-negative staphylococci (CNS) strains used in the study. MIC values of Corallopyronin A, methicillin resistance status, antimicrobial resistance patterns, and strain origin are shown

Nb	CNS strains	MIC CorA (mg/L)	Methicillin-resistance	Additional resistances
1	*S. capitis* CNS1	2	methS	Fos
2	*S. pettenkoferi* CNS2	2	methS	Ery, Cli, Fos, Fus, Rif
3	*S. capitis* CNS3	1	methS	FosR
4	*S. capitis* CNS4	1	methR	Gen, Fos
5	*S. epidermidis* CNS5	1	methS	-
6	*S. epidermidis* CNS6	1	methR	Tet, Ery, Cli, Fus, Lev
7	*S. epidermidis* CNS7	0.5	methS	Fus
8	*S. epidermidis* CNS8	0.5	methR	Gen, Tet, Ery, Cli, Fos, Lev, Rif
9	*S. epidermidis* CNS9	0.5	methR	Sxt
10	*S. epidermidis* CNS10	0.5	methS	Fus
11	*S. epidermidis* CNS11	0.5	methR	Ery, Cli, Lev
12	*S. lugdunensis* CNS12	0.5	methS	Pen, Tet
13	*S. haemolyticus* CNS13	0.5	methR	Oxa, Fos
14	*S. haemolyticus* CNS14	0.5	methR	Sul, Gen, Tet, Ery, Fos, Fus, Lev, Rif
15	*S. hominis* CNS15	0.5	methS	Ery, Fos
16	*S. capitis* CNS16	0.5	methR	Sulb, Cef, Gen, Ery, Cli, Fos, Lev
17	*S. caprae* CNS17	0.5	methR	Gen, Ery, Cli, Fos, Lev
18	*S. caprae* CNS18	0.5	methS	Ery, Fos, Fus
19	*S. haemolyticus* CNS19	0.5	methR	Sxt, Cef, Ery, Fos, Tei, Fus
20	*S. haemolyticus* CNS20	0.5	methS	Tet, Ery, Fos, Fus
21	*S. epidermidis* CNS21	0.5	methS	Ery, Fos, Fus
22	*S. epidermidis* CNS22	0.5	methR	Gen, Ery, Cli, Fos, Sxt, Lev, Rif
23	*S. epidermidis* CNS23	0.5	methR	Gen, Tet, Ery, Cli, Fus, Lev, Rif
24	*S. warneri* CNS24	0.5	methS	Fos, Fus
25	*S. pettenkoferi* CNS25	0.5	methS	Fos, Fus
26	*S. hominis* CNS26	0.5	methS	-
27	*S. epidermidis* CNS27	0.25	methR	Gen, Tet, Ery, Cli, Fos, Sxt, Tei, Fus, Lev, Rif
28	*S. epidermidis* CNS28	0.25	methR	Gen, Ery, Cli, Fos, Sxt, Fus, Lev, Rif
29	*S. epidermidis* CNS29	0.25	methR	Gen, Tet, Ery, Cli, Fos, Sxt, Lev, Rif
30	*S. epidermidis* CNS30	0.25	methS	Ery, Cli, Tei
31	*S. caprae* CNS31	0.25	methS	Fos, Fus
32	*S. haemolyticus* CNS32	0.25	methR	Ery, Fos, Tei, Fus
33	*S. haemolyticus* CNS33	0.25	methS	Tet, Cli, Fos
34	*S. haemolyticus* CNS34	0.25	methR	Sul, Cef, Gen, Ery, Cli, Fos, Sxt, Lev, Rif
35	*S. haemolyticus* CNS45	0.25	methR	Sul, Cef, Gen, Tet, Ery, Cli, Fos, Sxt, Lev, Rif
36	*S. haemolyticus* CNS36	0.25	methR	Sul, Cef, Gen, Tet, Ery, Cli, Fos, Sxt, Lev, Rif
37	*S. haemolyticus* CNS37	0.25	methS	Tet, Ery, Fos, Fus
38	*S. capitis* DSM20326 ATCC 27840*	1	methS	Tet, Fos
39	*S. epidermidis* ATCC 12228*	1	methS	Cli, Lin, Van, Tet, Fos, Fus
40	*S. haemolyticus* ATCC 29970*	0.5	methS	Fos
41	*S. warneri* ATCC 27836*	0.5	methS	Fos, Cli
42	*S. epidermidis* ATCC 14990**	0.5	methS	Tet
43	*S. hominis* ATCC 27844*	0.25	methS	Tet, Fos, Fus

Antibiotic abbreviations: *Tet* tetracycline, *Ery* erythromycin, *Fus* fusidic acid, *Cip* ciprofloxacin, *Cli* clindamycin, *Lev* levofloxacin, *Gen* gentamicin, *Sxt* trimethoprim-sulfamethoxazole, *Rif* rifampicin, *Van* vancomycin, *Tei* teicoplanin, *Oxa* oxacillin, *Lin* lincomycin. All clinical CNS strains were isolated after sonication of operative materials obtained from prosthetic joint infections

Type (*) or quality control strains (**) are marked with asterisks

The frozen isolates (- 80 °C) were freshly grown overnight on Columbia blood agar (Fisher Scientific, Illkirch, France) at 37 °C. Pre-cultures were inoculated with a single colony into 10 mL Muller-Hinton Broth in an incubator shaker at 37 °C.

### Antibiotics

CorA, a naturally occurring α-pyrone antibiotic (purity > 90%), was produced at the Helmholtz Centre for Infection Research using the heterologous producer strain *Myxococcus xanthus*, which contains the complete biosynthesis gene cluster for CorA [25]. In addition to CorA, various antibiotics were utilized in the checkerboard assays. These included acyldepsipeptide antibiotic-ADEP 2 (provided by H. Brötz-Oesterhelt, Tübingen), ceftobiprole (TOKU-E, Bellingham, WA, USA), ciprofloxacin (Sigma-Aldrich, St. Louis, MO, USA), clindamycin (Biomol, Hamburg, Germany), dalbavancin (Biomol, Hamburg, Germany), daptomycin (Cipla Europe NV, Antwerpen, Belgium), fosfomycin (Abcam, Cambridge, UK), linezolid (Panpharma, Luitré, France), oritavancin (Cayman Chemical, Ann Arbor, MI, USA), tigecycline (Pfizer, New York, NY, USA), and vancomycin (Dr. Eberth, Wesel, Germany). Each antibiotic was prepared according to the manufacturer's instructions to ensure optimal performance for the assays. Mueller–Hinton broth used for daptomycin checkerboard assays was supplemented with calcium (50 μg/mL Ca^2+^), in accordance with EUCAST recommendations.

Susceptibility testing was performed using the VITEK^®^ 2 system (bioMérieux, Marcy-l'Étoile, France) at the Institute of Medical Microbiology, Immunology and Parasitology, University Hospital Bonn (Germany).

### Evaluation of minimum inhibitory concentrations (MICs) and minimal bactericidal concentrations (MBCs) of CorA

The minimum inhibitory concentrations (MICs) and minimal bactericidal concentrations (MBCs) of CorA were determined using a standard broth microdilution method following EUCAST guidelines. The assays were conducted in sterile 96-well microplates using Mueller–Hinton (MH) broth (Oxoid, Thermo Scientific, Waltham, MA, USA) as a growth medium, with an inoculum density of 1-5 × 10^5^ CFU/ml. The MIC was defined as the lowest CorA concentration inhibiting visible bacterial growth after 24 h of incubation at 37 °C.

Stock solutions of CorA were prepared at a concentration of 1 mg/ml in dimethyl sulfoxide (DMSO) and stored in dark glass tubes with inserts at -80 °C. On the day of the experiment, a working solution of CorA (16 μg/ml MH, 2% DMSO) was prepared by diluting the stock solution in MH medium. For the MIC assay, 100 μl of the stock solution was added to the first well of each row in a 96-well plate, which had been pre-loaded with 100 µl MH, 2% DMSO in all wells. A twofold serial dilution was performed from wells 1 to 10. Well 11 served as a growth control, and well 12 contained medium with 1% DMSO (sterile control). A bacterial suspension was prepared by adjusting the optical density (OD_600_) to 0.1 and diluting it 1:100 in MH broth. Then, 100 μl of this suspension was added to each well (columns 1 to 12) to achieve the desired bacterial concentration in MH, 1% DMSO, with the highest concentration of 4 mg/l CorA in the first well. The plates were incubated at 37 °C for 24 h. The daily working solution (1250 μl) was sufficient for running the assay for four bacterial strains in parallel, each tested in triplicate.

The minimal bactericidal concentration (MBC) was determined by subculturing 100 μl from wells showing no visible bacterial growth in the MIC assay and two additional wells with higher concentrations of CorA. Aliquots and their dilutions were plated in triplicate on Columbia agar plates (Oxoid, Thermo Scientific) and incubated at 37 °C for 24 h. Small-colony variant (SCV) strains were incubated at 37 °C for 48 h. Control wells were plated as 1:1000 dilutions directly after inoculation to determine CFU counts of the inoculum. After incubation, the number of colonies was counted. The MBC was defined as the lowest concentration of CorA that resulted in a ≥ 99.9% reduction in the initial bacterial inoculum, following the methodology described by Pankey and Sabath (2004)[26].

### Time-kill assays

Time-kill assays were performed in the presence of 4 × MIC for five selected *S. aureus* strains: *S. aureus* HG001, *S. aureus* 6850, *S. aureus* ATCC 25923, *S. aureus* ATCC 25923, and *S. aureus* ATCC 33591. Overnight cultures of strains were inoculated in 40 mL of Mueller–Hinton (MH) media and incubated in flasks at 37 °C with continuous shaking until the culture reached an optical density (OD) of 0.3. Subsequently, 15 mL of the 0.3 OD bacterial culture was transferred to 150 mL Erlenmeyer flasks, and CorA was added at a concentration of 4 × MIC. The cultures were then incubated in an orbital shaker at 37 °C.

At hourly intervals from 1 to 5 h and again after 24 h, 100 µL samples were collected from both the control and the CorA-treated cultures. These samples were serially diluted by transferring 100 µL into 900 µL of MH broth. From each dilution (10^–1^ to 10^–7^), 100 µL was plated onto Columbia blood agar in triplicate. After 18 to 24 h of incubation, colonies were counted to determine CFU/mL and time-kill curves were generated to illustrate the reduction of CFU over time.

### Checkerboard assays

Checkerboard assays were performed to assess the activity of CorA in combination with other antibiotics against *S. aureus* USA300 and *S. aureus* 6850. MIC testing was carried out in two 96-well plates, following EUCAST guidelines. In one plate, CorA was serially diluted along the x-axis, while the second antibiotic was diluted along the y-axis in another plate.

For the combination test, 50 µL from each corresponding well of the two plates were combined into a new plate, generating combinations of CorA and the second antibiotic at varying concentrations, with a final volume of 100 µL in each well. Column 12 served as the control for CorA alone, while row H contained only the second antibiotic.

Bacterial suspensions were added to each well, bringing the final bacterial density to 5 × 10^5^ CFU/mL, and plates were incubated at 37 °C for 24 h. For evaluation, the fractional inhibitory concentration (FIC) index was calculated. The FIC index evaluates the effect of two antibiotics in combination relative to when used individually. The interaction was interpreted as synergistic if the FIC index was ≤ 0.5, additive if > 0.5 and ≤ 1, indifferent if > 1 and ≤ 4, and antagonistic if ≥ 4. The addition of 0.02% Tween 80 in assays involving dalbavancin and oritavancin did not affect bacterial growth or MIC values. The final DMSO concentration in all checkerboard assays did not exceed 1% (v/v).

This experiment was performed three times on separate days to ensure accuracy.

### Statistics and data analysis

All statistical analyses were conducted using RStudio (Version 2024.04.0 + 735, R Foundation for Statistical Computing, Vienna, Austria). Chi-square tests were used to compare MIC distributions between methicillin-resistant and methicillin-sensitive strains, due to non-normal data distribution. A p-value < 0.05 was considered statistically significant.

### Bacterial typing

Types in Tables 1, 2 derive from spa typing or PFGE patterns [27, 28], literature data or genomic data using MLST 2.0, (https://cge.food.dtu.dk/services/MLST-2.0/). The *S. aureus* Mu50 resistance profile was established using Resfinder 4.0 (http://genepi.food.dtu.dk/resfinder).

## Results

### Minimum inhibitory concentrations (MIC)

In our study, we assessed the minimum inhibitory concentrations (MIC), MIC_50_ and MIC_90_ of CorA against 73 strains of *Staphylococcus aureus* (including 2 stable SCV strains, *S. aureus* I10 [29] and *S. aureus* Ia48 [30]) and 43 coagulase-negative staphylococci (CNS). The collection included laboratory strains and clinical isolates from prosthesis, soft tissue and blood stream infections. Selected strains of *S. aureus* included strains representing different pan- and epidemic hospital-associated MRSA, community-associated MRSA, livestock-associated MRSA, two VISA strains with very diverse resistance profiles, and a strain collection from prosthetic joint infections comprising *S. aureus* and CNS, isolated from patients between 2013 and 2022 at University Hospital Bonn. All strains tested were found to be sensitive to CorA, demonstrating its efficacy; this was also true for rifampicin-resistant isolates and VISA strains.

For *S. aureus* strains, the MIC values ranged from 0.125 mg/L to 1 mg/L, indicating a promising susceptibility profile. Specifically, methicillin-resistant *S. aureus* (MRSA) had MIC values between 0.125 mg/L and 0.5 mg/L, whereas methicillin-sensitive *S. aureus* (MSSA) displayed a broader range from 0.25 mg/L to 1 mg/L (Tables 1, 2, 3). The MIC_50_ and MIC_90_ for *S. aureus* were 0.25 mg/L and 0.5 mg/L, respectively, for both MRSA and MSSA. Although the MIC_50_ values were equal for MRSA and MSSA, MRSA exhibited significantly lower MIC values for CorA (p = 0.0382), highlighting its greater efficacy against methicillin-resistant strains.

Notably, two stable small colony variants of *S. aureus*, representing MRSA (*S. aureus* Ia48) and MSSA (*S. aureus* I10), had an MIC value of 0.25 mg/L indicating that their susceptibility to CorA was within the lower range observed for *S. aureus*, despite their enhanced resistance during antibiotic therapy with other agents (Tables 1, 2, 3) [30, 31].

Conversely, CNS strains exhibited higher MIC values, ranging from 0.25 mg/L to 2 mg/L (Table 4). The MIC_50_ and MIC_90_ for CNS were 0.5 mg/L and 1 mg/L, respectively, indicating a higher resistance profile than *S. aureus*. Methicillin-resistant CNS strains showed lower MICs for CorA than methicillin-sensitive strains (p = 0.018). The MIC values for methicillin-resistant CNS ranged from 0.25 mg/L to 1 mg/L, while two methicillin-sensitive CNS strains had MIC values of 2 mg/L.

The MIC distribution of CorA was analyzed against clinical isolates (hospital strains from prosthesis, soft tissue and bloodstream infections) and all tested isolates of *S. aureus* and coagulase-negative staphylococci (CNS) to assess differences in susceptibility patterns. For *S. aureus* isolates, tissue/blood isolates exhibited a similar MIC distribution to the broader dataset (Tables 1, 2, 3).

### Time-kill assays and minimum bactericidal concentrations (MBC)

**Fig. 1:**
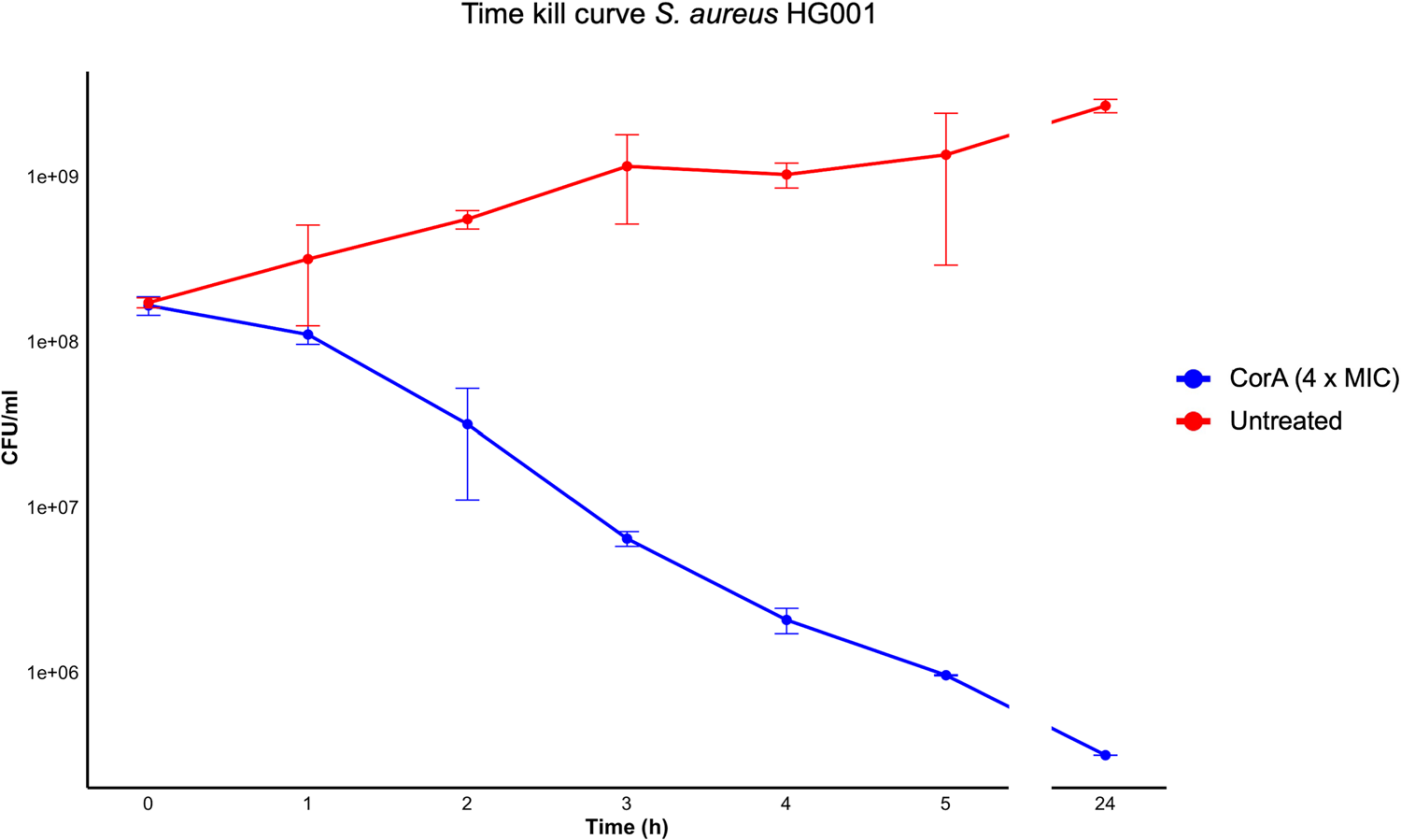
Time-kill curve of *Staphylococcus aureus* HG001 treated with CorA (4 × MIC) over 24 h. Bacterial viability decreased from 1.5 × 10^8^ CFU/mL to 3.3 × 10^5^ CFU/mL, indicating a pronounced bactericidal effect. Untreated control cultures showed continued growth over the same period. The Y-axis is plotted on a logarithmic scale. The standard deviation was calculated from triplicate measurements

The kinetics of bacterial killing were examined using time-kill assays, which provide a dynamic view of the antimicrobial activity of a CorA over time. In our study, we monitored the bacterial count at seven time points within 24 h to determine the effect of the CorA. Time-kill curves were generated in the presence of 4 × MIC for five different *S. aureus* strains: *S. aureus* HG001 (Fig. 1), *S. aureus* 6850, *S. aureus* ATCC 25923, *S. aureus* COWAN1, ATCC 12598, and *S. aureus* ATCC 33591 (Supplementary Fig. 1).

In the presence of 4 × MIC, all five tested strains exhibited an average of 1.6 to 3 logs reduction in viable bacterial cell counts within 24 h. Among the strains tested, strain *S. aureus* HG001 showed the greatest reduction in cell count from 1.5 × 10^8^ to 3.3 × 10^5^ after 24 h, highlighting its high susceptibility to CorA (Fig. 1). In contrast, the bacteria increased in the untreated control samples over time.

**Fig. 2:**
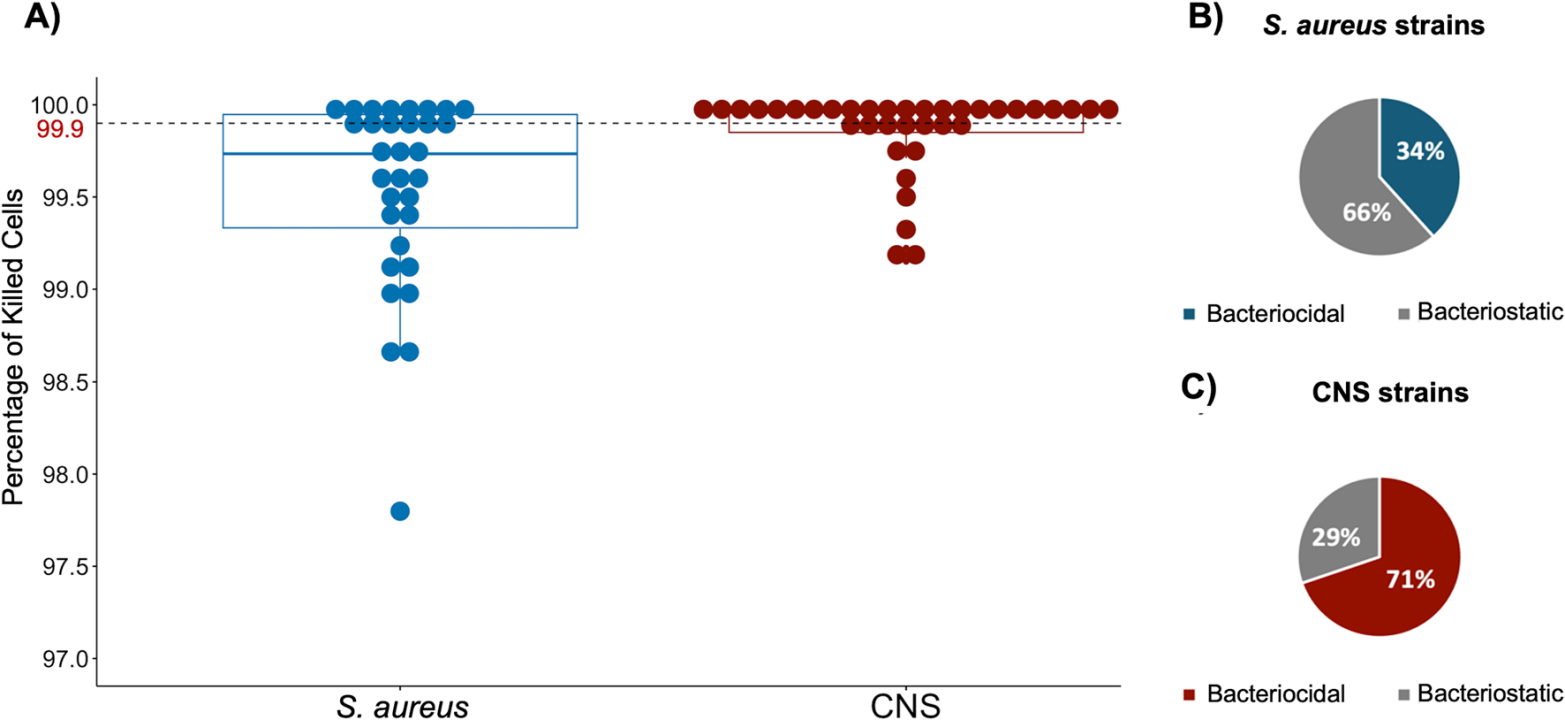
**A** Percentage of *Staphylococcus aureus* and coagulase-negative staphylococci (CNS) cells killed after treatment with Corallopyronin A at 4 × MIC. Each dot represents an individual strain. The dashed line at 99.9% indicates the bactericidal threshold. The boxplot illustrates the median, interquartile range, and variability excluding outliers, which are shown as individual dots. The Y-axis is truncated at 97% for clarity; full data are provided in Supplementary Table 1.** B** Proportion of *S. aureus* strains exhibiting bactericidal (blue) or bacteriostatic (gray) responses. **C** Proportion of CNS strains showing bactericidal (red) or bacteriostatic (gray) activity following treatment

To further evaluate whether CorA can be classified as a bactericidal antibiotic, we determined the minimum bactericidal concentrations (MBCs) for 70 strains. The MBC is the lowest concentration of an antimicrobial agent required to kill 99.9% of the initial bacterial population. If more than 0.1% of the inoculum remained viable after 24 h of incubation in the wells containing the MIC, 2 × MIC, or 4 × MIC of CorA, the result was recorded as bacteriostatic [40]. According to these criteria, bactericidal activity of CorA was confirmed in 34% of the tested *S. aureus* strains and 71% of the CNS strains (Fig. 2, Supplementary Table 3). A 99% reduction of CFU was found for 97.37% of all tested CNS and 87.50% of all *S. aureus* strains.

Specifically, CorA exhibited bactericidal activity in 26% of methicillin-resistant *S. aureus* (MRSA) strains and 78% of methicillin-resistant CNS strains (Supplementary Tables 4 & 5). Furthermore, CorA had a bactericidal effect on 55% of MSSA and 65% of methicillin-sensitive CNS strains (Supplementary Tables 4 & 5). The Chi-square test results indicated a significant difference in the bactericidal efficacy of CorA between methicillin-resistant and methicillin-sensitive strains (p = 0.03), considering both CNS and *S. aureus*. This highlights that the effectiveness of CorA varies depending on methicillin resistance status and between *Staphylococcus aureus* and coagulase-negative staphylococci. Within the group of coagulase-negative staphylococci, no systematic species-specific differences were assessed.

The higher bactericidal activity against CNS compared to *S. aureus* indicated a good efficacy of CorA towards coagulase-negative staphylococci, which are often involved in prosthetic device infections. In summary, the time-kill assays and MBC determinations provide evidence of the bactericidal properties of CorA, particularly against methicillin-resistant strains of both *S. aureus* and CNS. These findings support the potential use of CorA as a potent antimicrobial agent in treating infections caused by these resistant strains.

### Checkerboards assays

To evaluate the synergistic potential of CorA in combination therapy, checkerboard assays were conducted with CorA against *Staphylococcus aureus* USA 300 (community-associated MRSA, source NARSA, CC8) and MSSA strain *S. aureus* 6850 (a clinical isolate from a joint infection, ST50) [41].

The selected antibiotics included an acyldepsipeptide antibiotic (ADEP2), ceftobiprole, ciprofloxacin, clindamycin, dalbavancin, daptomycin, fosfomycin, linezolid, oritavancin, tigecycline and vancomycin. Given that *S. aureus* USA 300 is resistant to clindamycin, ciprofloxacin, and fosfomycin, checkerboard assays were not performed for these antibiotics.

CorA exhibited additive effects when combined with the newer glycopeptides, dalbavancin and oritavancin (Table 5). Additionally, the checkerboard assays yielded a fractional inhibitory concentration index (FICI) of 1 for combinations involving vancomycin, clindamycin, and linezolid, indicating an additive interaction. Other combinations, such as those with ceftobiprole, tigecycline, daptomycin, and fosfomycin, were indifferent.

**Table 5:** Fractional inhibitory concentration (FIC) of CorA combined with different antibiotics. Checkerboard assays were conducted for MRSA strain *S. aureus* USA 300 and MSSA strain *S. aureus* 6850. Interpretation of FIC index values: synergistic (≤ 0.5), additive (> 0.5 to 1), indifferent (> 1 to 4), and antagonistic (≥ 4). MIC values for CorA are listed for each strain. No antagonistic interactions were observed

Strain ID	MIC CorA (μg/mL)	Antibiotic	FIC index	Effect
*S. aureus* USA 300	0.25	Dalbavancin	0.756	Additive
		Linezolid	1	Additive
		Vancomycin	1	Additive
		Oritavancin	1.125	Indifference
		ADEP 2	1.126	Indifference
		Tigecycline	1.252	Indifference
		Daptomycin	1.5	Indifference
		Ceftobiprole	2	Indifference
*S. aureus* 6850	0.25 - 0.5	Oritavancin	0.625	Additive
		Dalbavancin	0.756	Additive
		ADEP 2	1	Additive
		Clindamycin	1	Additive
		Vancomycin	1	Additive
		Ceftobiprole	1.125	Indifference
		Linezolid	1.5	Indifference
		Tigecycline	1.5	Indifference
		Ciprofloxacin	1.5	Indifference
		Daptomycin	1.5	Indifference
		Fosfomycin	2	Indifference

## Discussion

This study provides a detailed evaluation of the antimicrobial properties of CorA against a diverse collection of *S. aureus* and coagulase-negative staphylococci (CNS). The dataset included 116 strains for MIC determination and 70 strains for MBC evaluation, encompassing hospital-derived clinical isolates, mostly from joint and prosthesis infections, and laboratory strains (Supplementary Table 1). This diversity spans methicillin-resistant and methicillin-sensitive strains of *S. aureus* and CNS, vancomycin-intermediate *S. aureus* (VISA), community-associated MRSA, livestock-associated MRSA, and small colony variants (SCVs). The inclusion of this diverse collection of staphylococcal strains enabled a comprehensive assessment of the potential of CorA for treating challenging infections, including osteomyelitis and periprosthetic joint infections.

### Antimicrobial efficacy and methicillin resistance

A significant finding in this study is the higher susceptibility to CorA of methicillin-resistant strains compared to methicillin-sensitive ones. Methicillin-resistant *S. aureus* and CNS exhibited significantly lower MIC values than their methicillin-sensitive counterparts (p = 0.038 for MICs, p = 0.03 for MBCs). This higher susceptibility in methicillin-resistant strains might be attributed to differences in cellular physiology, potentially related to the fitness costs of methicillin-resistance mechanisms. In another study, clinical MRSA were also more susceptible to rifampicin compared to MSSA [42]. Interestingly, *Nikolic *et al. (2023) confirmed differences in protein profiles between methicillin-sensitive and -resistant clinical strains of *S. aureus*, with a significantly higher level of polyribonucleotide nucleotidyltransferase in MSSA, an enzyme that is involved in the breakdown of mRNA [40]. This enzyme is part of the staphylococcal RNA degradosome [43]. In *E. coli,* the homologous PNPase is involved in rifampicin-induced rRNA degradation, leading to a strong decrease of ribosomal RNA and setting the cells to a reversible resting state during rifampicin treatment [44], thus dealing with rifampicin stress. Future studies should explore the molecular basis of this differential susceptibility to optimize the clinical application of CorA.

The study also highlights the activity of the inhibitor against SCVs, a phenotype often associated with chronic and biofilm-related infections. SCVs are characterized by their slow growth and enhanced persistence in host environments [45, 46]. Despite these challenges, both SCVs (*S. aureus* I10 [29] and *S. aureus* Ia48 [30]) exhibited MIC values within the lower range observed for *S. aureus*, suggesting strong antimicrobial efficacy against these persistent forms. This finding is particularly relevant for infections such as prosthetic joint infections (PJIs), where SCVs play a key role in treatment failures and recurrent infections [6, 7]. However, only two stable, genetically engineered SCV isolates could be tested by microdilution, because spontaneous, clinical isolates are often unstable in liquid culture. It has been shown that even a single revertant cell in the inoculum of 1-5 × 10^5^ cells/ml would be able to overgrow the SCV during the 48 h incubation time and falsify results [47]. Therefore, the observed activity of CorA against SCVs should be considered preliminary.

Similar observations have been reported in previous studies investigating the antimicrobial efficacy of other antibiotics against SCVs. For instance, daptomycin demonstrated potent pharmacodynamic activity against *S. aureus* SCVs (*S. aureus* Ia48), despite the inherent antibiotic tolerance exhibited by these phenotypes during treatment, which may be due to their enhanced intracellular survival in macrophages [25, 30]. CorA is very effective against intracellular bacteria [20–22, 48, 49] and this result highlights its therapeutic promise for addressing persistent intracellular infections that are refractory to conventional treatments.

### Bactericidal activity and time-kill kinetics

A bactericidal activity would be particularly relevant for managing biofilm-associated infections, where completely eradicating bacteria is critical. Biofilms, often formed by CNS and SCVs in PJIs, create environments that are highly tolerant to conventional antibiotics.

The bactericidal effects observed in the study are promising. Methicillin-resistant strains showed a higher proportion of bactericidal outcomes compared to methicillin-sensitive strains, as demonstrated by MBC results and time-kill assays (Supplementary Table 4). In particular, methicillin-resistant CNS strains were susceptible to the bactericidal activity of CorA (78% of all tested methicillin-resistant CNS strains (Supplementary Table 5)). Similarly, methicillin-resistant *S. aureus* strains demonstrated a higher percentage of bactericidal effects compared to MSSA strains. The time-kill assays further support these findings, showing significant reductions in viable bacterial counts within 24 h. However, since bactericidal effects could not be demonstrated for all strains, these results place CorA action on the brink between bactericidal and bacteriostatic. The reason for this effect is still unclear since the consequences of inhibiting RNA-polymerase that result in the bactericidal effects of CorA have not yet been elucidated and will be the focus of further studies. For rifampicin, which also targets RNA-polymerase, a clear bactericidal activity is seen for *M. tuberculosis* [50], whereas its effect on *E. coli* is less lethal [51], indicating that different killing mechanisms may be involved in different species.

### Synergy with existing antibiotics

The evaluation of this agent in combination with 11 antibiotics revealed additive effects with glycopeptides, including dalbavancin and oritavancin. These findings are particularly promising, as glycopeptides are frequently used to treat biofilm-associated infections due to their efficacy against slow-growing bacterial populations. Notably, a study by *Baldoni* et al. (2013) demonstrated that dalbavancin, when combined with rifampicin, improved the killing of planktonic MRSA compared to dalbavancin alone, though time-kill curves indicated neither synergism nor antagonism in the interaction [52]. This underscores the potential of combination therapies to enhance antimicrobial efficacy, even when specific interactions are not strictly synergistic.

Additionally, the absence of antagonistic interactions with clinically relevant antibiotics, including vancomycin and linezolid, supports the potential integration of this inhibitor into combination therapy regimens. Although most interactions were classified as additive rather than synergistic, this finding remains clinically relevant, as additive combinations without antagonism may still enhance therapeutic efficacy and help limit the emergence of resistance in staphylococcal infections [3].

The findings presented here demonstrate the potential of the RNA polymerase inhibitor CorA to address several unmet clinical needs. Its efficacy against methicillin-resistant strains and SCVs underscores its therapeutic potential for multidrug-resistant staphylococcal infections, including PJIs and intracellular infections. Further research should focus on transcriptomic and proteomic analyses to elucidate the underlying mechanisms contributing to the higher susceptibility of MRSA compared to MSSA, particularly concerning differences in gene expression, metabolic adaptations, and potential interactions with RNA polymerase.

A limitation of this study is that all experiments were performed using planktonic bacteria, and CorA activity against biofilm-embedded staphylococci was not assessed. Future studies should therefore evaluate CorA in biofilm models to further explore its therapeutic potential in biofilm-associated infections.

## Conclusion

This study highlights the robust antimicrobial activity of CorA against a diverse collection of *Staphylococcus aureus* and coagulase-negative staphylococci (CNS) strains, including methicillin-resistant strains and a limited number of tested small colony variants (SCVs). The strain collection included isolates from both hospital strains from prosthesis, soft tissue and bloodstream infections and laboratory isolates, representing a wide range of resistance profiles. The significant differences in susceptibility between methicillin-resistant and methicillin-sensitive strains, together with demonstrated bactericidal activity in a subset of isolates and additive interactions with existing antibiotics, underscore the therapeutic potential of CorA.

These findings provide a solid foundation for further preclinical evaluations aimed at developing CorA as a therapeutic option for multidrug-resistant staphylococcal infections, including those associated with prosthetic joint infections, and warrant future studies addressing its activity in biofilm models.

## Supplementary Information

Below is the link to the electronic supplementary material.

Supplementary file1 (XLSX 15 KB)

Supplementary file2 (DOCX 143 KB)

Supplementary file3 (DOCX 19 KB)

## Data Availability

No datasets were generated or analysed during the current study.
