# Supplementary material for: Corallopyronin A exhibits potent activity against staphylococci including MRSA and isolates from prosthetic infections
Source: Infection. 2026 Mar 12;54(3):1369–81. doi: 10.1007/s15010-026-02760-8 (PMC13323117; doi:10.1007/s15010-026-02760-8)
Supplement: Supplementary file 2 — Supplementary file2 (DOCX 143 KB) [file 15010_2026_2760_MOESM2_ESM.docx]

**
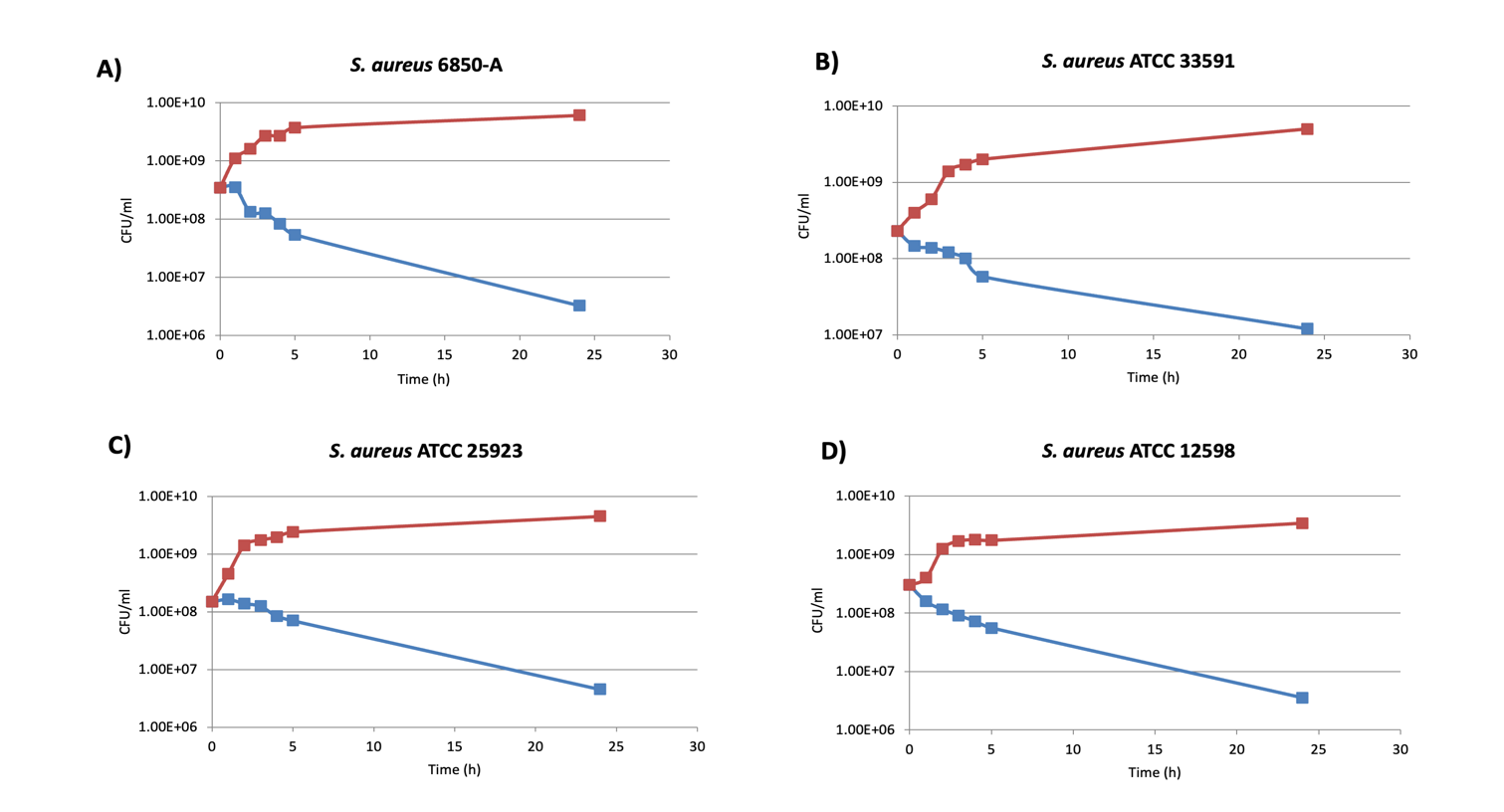
**

**Supplementary Figure 1:** Time-kill kinetics of Corallopyronin A against four *Staphylococcus aureus* strains (A) *S. aureus* 6850A, (B) ATCC 33591, (C) ATCC 25923, and (D) ATCC 12598). Bacterial cultures were exposed to CorA at 2 µg/mL (blue curves), corresponding to 4 × MIC for all strains (Table 1 & 2). Untreated control cultures are shown in red. Viable counts (CFU/mL) were determined at the indicated time points over 24 h of incubation at 37 °C. The data shown are representative of three independent experiments.
