# Supplementary material for: Corallopyronin A exhibits potent activity against staphylococci including MRSA and isolates from prosthetic infections
Source: Infection. 2026 Mar 12;54(3):1369–81. doi: 10.1007/s15010-026-02760-8 (PMC13323117; doi:10.1007/s15010-026-02760-8)
Supplement: Supplementary file 3 — Supplementary file3 (DOCX 19 KB) [file 15010_2026_2760_MOESM3_ESM.docx]

**Supplementary Table 2:** Distribution of *S. aureus* and CNS strains according to MICs

| **MICs** | ***S. aureus*** | **CNS** |
| --- | --- | --- |
| MIC - range (mg/L) | 0.125 - 1 | 0.25 - 2 |
| MIC_50_ (mg/L)  (i.e., 50 % of tested strains are inhibited) | 0.25 | 0.5 |
| MIC_90_ (mg/L) | 0.5 | 1 |
| MICs small colony variants  (2 stable strains), both strains | 0.25 | n.d.* |

* not determined

**Supplementary Table 3:** Distribution of *S. aureus* and CNS strains according to MBC

| **MBCs** | *S. aureus* | CNS |
| --- | --- | --- |
| Bactericidal action (no. of strains) | 11 | 27 |
| Bacteriostatic action (no. of strains) | 21 | 11 |

**Supplementary Table 4:** Number of MRSA and MSSA strains according to MBC results.

|  | **MRSA** | **MSSA** |  |
| --- | --- | --- | --- |
| Bacteriocidal | 6 | 5 |  |
| Bacteriostatic | 17 | 4 |  |
|  | 23 | 9 |  |
|  |  |  |  |
|  |  |  |  |
| **%** | **MRSA (%)** | **MSSA (%)** |  |
| Bacteriocidal | 26.08 | 55.55 |  |
| Bacteriostatic | 73.91 | 44.44 |  |

**Supplementary Table 5:** Distribution of Methicillin-resistant and Methicillin-sensitive NCS strains according to MBCs results.

|  | **MBC** | |  |
| --- | --- | --- | --- |
|  | **Meth-R** | **Meth-S** |  |
| **CNS** | 18 | 20 |  |
| Bacteriocidal | 14 | 13 |  |
| Bacteriostatic | 4 | 7 |  |
|  |  |  |  |
|  |  |  |  |
| **% CNS** | **Meth-R** | **Meth-S** |  |
| Bacteriocidal | 77.77 | 65 |  |
| Bacteriostatic | 22.22 | 35 |  |
